# “Effect of Palatal Metal Collar's Height on Fracture Resistance of Single Metal-Ceramic Crowns”

**DOI:** 10.1155/2022/8290538

**Published:** 2022-10-07

**Authors:** Nour Aburaisi, Haitham Elbishari

**Affiliations:** ^**1**^ Restorative Dentist, Dr. Sulaiman Al Habib Hospital, Khobar, Saudi Arabia; ^**2**^ Department of Prosthodontics, Hamdan Bin Mohammed College of Dental Medicine (HBMCDM), Mohammed Bin Rashid University of Medicine and Health Sciences (MBRU), Dubai, UAE

## Abstract

**Objectives:**

The purpose of this study is to evaluate the effect of the palatal metal collar height on the fracture resistance of metal-ceramic crowns.

**Materials and Methods:**

A maxillary premolar typodont tooth was prepared and scanned to fabricate 48 metal analogs. The analogs were then scanned to fabricate metal copings divided into 4 groups according to palatal metal collar height as follows; (C0): 0 mm, (C1): 1.0 mm, (C2): 1.5 mm, and (C3): 2.0 mm. After a standard application of pressed ceramic, each crown was cemented onto its corresponding metal tooth analog. The crown-analog assembly was subjected to a sequence of thermal stressing for 5,000 cycles. A universal testing machine applied controlled loads to the crowns until fracture. Examination under a stereomicroscope determined the failure mode. A scanning electron microscope (SEM) was used to examine fracture. Load to failure data was analyzed using ANOVA followed by Tukey HSD (*P* ≤ 0.05).

**Results:**

ANOVA statistics revealed that groups with a palatal metal collar presented significantly higher failure loads when compared to the collarless group (*P* < 0.0001). Difference in failure loads between 1.5-mm and 2.0-mm palatal metal collar height were not statistically significant (*P* = 0.935). There were no significant differences detected among the groups in terms of failure mode.

**Conclusions:**

The height of the palatal metal collar has an effect on the fracture resistance of the metal-ceramic crowns. *Clinical Relevance*. The incorporation of a palatal collar with a predetermined height is essential to reduce the mechanical failure of metal-ceramic crowns.

## 1. Introduction

Although all-ceramic restorations have been used primarily in the anterior region because of their optimum esthetics and light transmission qualities [1], metal-ceramic restorations are still used extensively in dental practice and have been considered the “gold standard” [2, 3]. In addition to their superior physical properties and acceptable esthetics [4–9], metal-ceramic restorations offer several advantages such as abrasion resistance, color stability, and insolubility in oral fluids [10]. However, when the porcelain is applied in thin sections such as in the cervical third of the crown, esthetics may be compromised. This is due to poor light transmission and increased light reflectivity from the opaque porcelain that masks the underlying metal substructure [9, 11–13]. Furthermore, light grey discoloration of tissue surrounding the margin may result [9, 14]. To overcome these problems, many dental laboratory technicians tend to reduce the metal collar to a fine line prone to distortion during the ceramic firing cycles. Other attempts include masking the metal with ceramic, but this resulted in overcontouring which has adverse periodontal effects [15].

Elimination of the metal facially became an area of interest [16, 17]. The introduction of various techniques to fabricate the porcelain labial margin [18–20] provided a significant improvement in the esthetics of metal-ceramic restorations [9] with marginal accuracy comparable to metal margins [21–23] and less bacterial plaque accumulation [24]. However, light transmission properties were not greatly enhanced in the cervical portion of the restoration due to the failure of incidental light transmission through the entire body of the tooth. This is mainly due to the presence of the palatal metal margin [25]. This led to the introduction of the collarless metal-ceramic crown and the elimination of the metal collar [9, 15]. Although fabrication was technique sensitive, translucency and esthetic outcome were optimum [25].

Previous studies aimed mainly to identify the influence of the presence or absence of a 360° metal collar on the fracture resistance of single metal-ceramic crowns [9, 15]. Most framework design modifications were established facially [7, 9, 15]. Michalakis and colleagues compared the fracture resistance of metal-ceramic crowns with metal margins to that of metal-ceramic crowns with circumferential ceramic margins after exposure to masticatory simulation by cyclic loading. Their study concluded that metal-ceramic restorations with metal margins required significantly greater loads to fracture than collarless metal-ceramic restorations [9]. These findings agreed with the results of Goodacre et al., [15] who concluded that crowns with porcelain margins are less rigid than those with metal margins. However, these results were in contrast with the findings of Gardner et al., [7]. Gardener concluded that the required load to fracture porcelain from crowns with facial porcelain margins was greater than that for crowns with metal margins. Furthermore, modifications of the metal framework on premolars [26] and molars [27] were evaluated concluding that these modifications increased the strength of metal-ceramic crowns [26] while other studies concluded that such modifications were not significant [27].

Collarless metal-ceramic restorations are less resistant to fracture than restorations with 360° metal margin, yet more esthetic. The influence of the palatal metal collar height on mechanical strength has not been yet investigated. Therefore, the purpose of this study was to evaluate the effect of the palatal metal collar height on the fracture resistance of metal-ceramic single crowns. The null hypothesis was that different palatal metal collar height would not affect the fracture resistance of metal-ceramic single crowns.

## 2. Material and Methods

**Figure 1:**
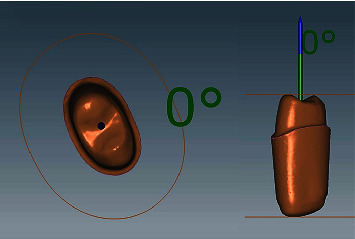
3D Scanned images of prepared tooth using ceramill mind CAD software (Amann girrbach, Vorarlberg, Austria).

**Figure 2:**
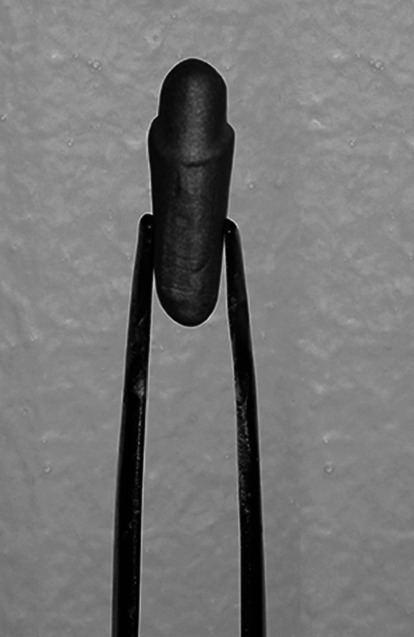
Metal analog of prepared right maxillary premolar.

All materials, with corresponding manufacturer details, used in this laboratory study are illustrated in Table1. A typodont (Dental model Ag3, Frasco, North Carolina, USA) maxillary right first premolar was prepared to receive a full coverage metal-ceramic restoration. A 2-mm occlusal reduction with a functional cusp bevel was accomplished, and a uniform, 2 planes, axial reduction was completed. The preparation of the finish line included a chamfer with rounded internal angles [28]. The amount of reduction was verified using a silicone putty index (Hydroise fast set putty, Zhermack, Badia Polesine, Italy). A lab scanner (Ceramill map 400 scanner, Amann Girrbach, Vorarlberg, Austria) was then used to capture digital images (Figure 1) of the prepared tooth to fabricate 48 metal analogs (Ceramill Sintron Chrome Cobalt (CoCr), Amann Girrbach, Vorarlberg, Austria), using CAD/CAM Ceramill Motion 2 milling unit (Amann Girrbach, Vorarlberg, Austria) (Table 2) [29]. Following sintering procedures (Ceramill Argotherm 2, Amann Girrbach, Vorarlberg, Austria), the analogs were inspected for any positive surface irregularities which were removed using a carbide fissure bur (NTI Finishing Bur, Kerr, California, USA) (Figure 2).

**Figure 3:**
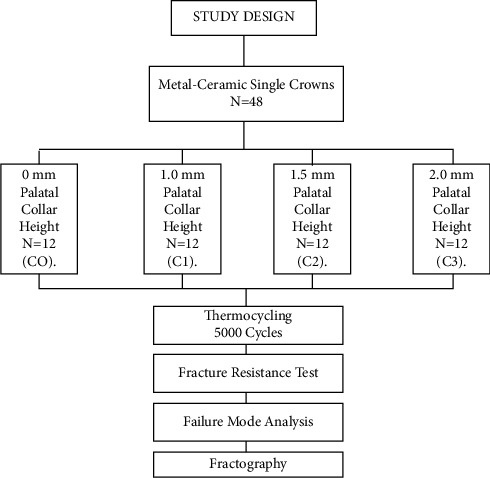
Study design. Figure shows the groups, number of crowns in each group and number of samples analyzed. (*N* = sample size).

**Figure 4:**
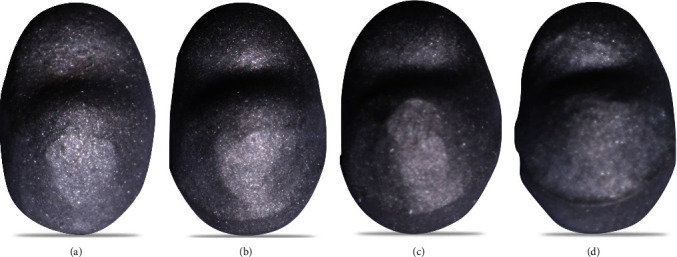
(a) Metal coping with 0 mm palatal metal collar in height or collarless. (b) Metal coping with 1.0 mm palatal metal collar in height. (c) Metal coping with 1.5 mm palatal metal collar in height. (d) Metal coping with 2.0 mm palatal metal collar in height.

The metal tooth analogs were then scanned to fabricate metal copings. Following the acquisition of digital data, 4 different designs of the metal coping according to the collar height were created using the Ceramill Mind CAD Software (Amann Girrbach, Vorarlberg, Austria) (Figure 3). The thickness of the metal coping was kept at 0.5 mm for all samples with a 1-mm thick palatal collar extending 1 mm to the proximal surfaces. All 48 copings were dry-soft milled in Ceramill Sintron CoCr using CAD/CAM Ceramill Motion 2 milling unit (Amann Girrbach, Vorarlberg, Austria). Following sintering procedures (Ceramill Argotherm 2, Amann Girrbach, Vorarlberg, Austria), metal copings were inspected to ensure design accuracy and the presence of any surface irregularities, which were removed using a carbide fissure bur (NTI Finishing Bur, Kerr, California, USA) and abraded externally and internally with 50-*µ*m aluminum oxide particles under 3 Kg/cm^2^ pressure (Basic Classic Dental Sandblaster, Remfert, Illinois, USA) (Figures 4(a)–4(d)). Metal copings were then placed on their corresponding metal analogs to ensure proper fit and accuracy.

A layer of ceramic bonding (Crea Alloy Bond, Creation Wili Geller, Meiningen, Austria) was applied to the metal copings prior to ceramic application as instructed by the manufacturer. The applied bonding layer was fired at 450°C to 980°C with a heat rate of 55°C/min (Programat EP 5010, Ivoclar Vivadent, Schann, Liechtenstein) and a holding time of 2 minutes at the end of the firing cycle. Two coats of opaque ceramic (IPS-InLine® Opaquer, Ivoclar Vivadent, Schann, Liechtenstein) were then applied and fired at 450°C to 930°C with a heat rate of 100°C/min (Programat EP 5010, Ivoclar Vivadent, Schann, Liechtenstein) and a holding time of 2 minute at the end of the firing cycle. Ceramic application for all specimens was accomplished by the same operator.

**Figure 5:**
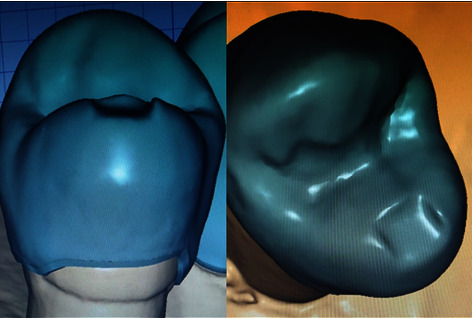
Full contoured maxillary right first premolar design incorporating a half-rounded 2-mm × 0.5-mm indentation on the palatal cusp in ceramill mind CAD software (Amann girrbach, Vorarlberg, Austria).

The crowns with opaque ceramic were scanned to create and design a full contoured maxillary right first premolar incorporating a half-rounded 2-mm × 0.5-mm indentation on the palatal cusp in Ceramill Mind CAD Software (Amann Girbach, Vorarlberg, Austria) and then imported as digital data to 3D Sprint Ceramill Software (Amann Girbach, Vorarlberg, Austria) (Figure 5). The resin patterns (Next Dent Cast, Amann Girbach, Vorarlberg, Austria), were 3D printed using Next Dent 5100 Ceramill (Amann Girbach, Vorarlberg, Austria). The patterns were carefully inspected for any visible resin residue and postcured in an ultraviolet furnace (Next Dent LC 3D Print Box, (Amann Girbach, Vorarlberg, Austria) for 5 minutes as recommended by the manufacturer. After verification of the thickness of the resin patterns using a thickness gauge, the resin patterns were fitted on the specimens and sealed with cervical wax (Renfert, Illinois, USA).

The metal copings with the resin patterns were invested using Bellavest SH (Bego, Bremen, Germany) investment material following the manufacturer's instructions. The press-over metal technique was used to build up the ceramic (IPS-InLine® POM, Ivoclar Vivadent, Schann, Liechtenstein). The investment ring was then transferred to the press oven and fired at 450°C to 930°C (Programat EP 5010, Ivoclar Vivadent, Schann, Liechtenstein) with a heat rate of 100°C and a holding time of 2 minutes. Then, each investment ring was divested (Basic Classic Dental Sandblaster, Renfert, Illinois, USA), placed in an ultrasonic cleaner (BioSonic UC125, Coltene/Whaledent, Altstatten, Switzerland) and finished and polished (Dentsply finishing and polishing kit, Dentsply Sirona, New York, USA). A glaze layer was then applied on the polished crowns (IPS Inline® Glaze, Ivoclar Vivadent, Schann, Liechtenstein) and fired at 450°C to 850°C (Programat EP 5010, Ivoclar Vivadent, Schann, Liechtenstein) with a heat rate of 100°C and a holding time of 2 minutes. The internal surfaces of the copings were carefully inspected and internally abraded with 50-*µ*m aluminum oxide particles under 3 Kg/cm^2^ pressure (Basic Classic Dental Sandblaster, Renfert, Illinois, USA).

A silicone abutment analog (DMG O-Bite, New Jersey, USA) was fabricated to ensure even cement thickness. Glass Ionomer luting cement (Ketac Cem Aplicap, 3M ESPE, Minnesota, USA) was used for cementation of the crowns following the manufacturer's instructions. After placing the cement in the fitting surface of the crown, the silicone abutment analog was inserted, and excess cement was removed using a cotton pellet. The silicone analog was removed, and the crown was seated over the metal analog resembling the prepared tooth using finger pressure maintained for 3 minutes at room temperature. All crown cementations were made by the same operator.

Following cementation procedures, the cemented crowns were stored in an incubator (Incubator I, Memmert GmbH, Schwabach, Germany) at room temperature 37°C for 24 hours. The cemented crowns were subjected to a sequence of thermal stressing between low (5°C) and high (55°C) temperature environments for a total number of 5,000 cycles according to the International Standards Organization (ISO) 10477 recommendations [30]. After thermocycling (Thermocycler THE-1100/THE-1200, SD Mechatronik, Feldkirchen-Westerham, Germany), all samples were examined for any ceramic cracks or fracture under a stereomicroscope with 35x magnification (Leica EZ4 stereomicroscope, Leica, Wetzlar, Germany).

**Figure 6:**
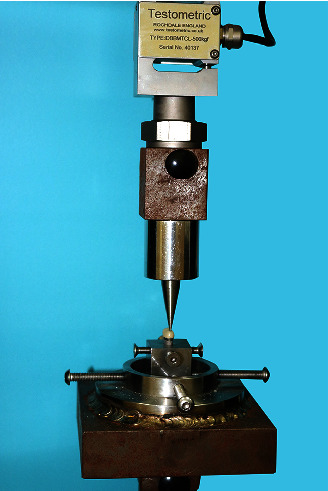
Metal tooth analog-crown-metal jig assembly mounted on universal testing machine (Testometric M350-5CT, Rochdale, United Kingdom).

**Figure 7:**
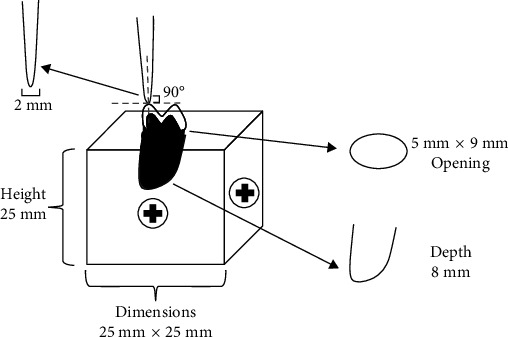
Schematic diagram of metal tooth analog-crown-metal jig assembly.

The crown-analog assembly for each specimen was mounted on a universal testing machine (Universal Testing Machine M350-5CT, Testometric, Rochdale, United Kingdom) using a customized metal jig manufactured to secure the sample on a metal base with its top surface 2 mm below the margin of the crown. A 2-mm wide, rounded end stainless steel rod attached to the upper member of the universal testing machine was used to apply controlled loads at a crosshead speed of 2.5 mm/min until fracture of the ceramic occurred (Figures 6 and 7). The load to fracture of all the samples was recorded in Newtons (N).

**Figure 8:**
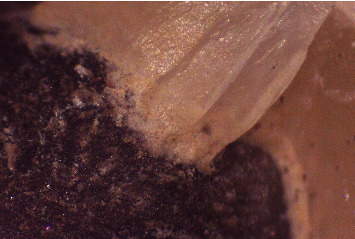
Stereomicroscopic images of the fractured crowns showing combined adhesive-cohesive mode of failure at 35x magnification.

**Figure 9:**
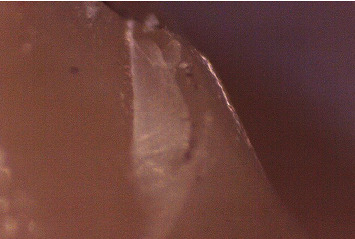
Stereomicroscopic images of the fractured crowns showing cohesive mode of failure at 35x magnification.

**Figure 10:**
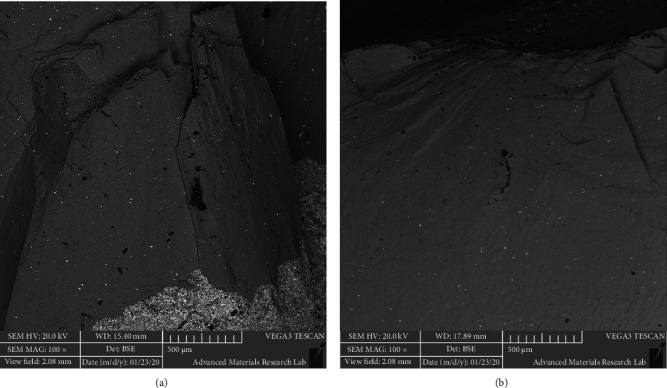
(a) Scanning electron microscopic images of the fractured metal-ceramic crowns at 100x magnification showed the presence of mirrors, hackles and wake hackles. (b) Scanning electron microscopic images of the fractured metal-ceramic crowns at 100x magnification showed the presence of mirrors, hackles and wake hackles.

After the fracture, the specimens were examined under a stereomicroscope (Leica EZ4 stereomicroscope, Leica, Wetzlar, Germany) under 35x magnification to assess whether the failure was of a cohesive, adhesive, or a combined cohesive-adhesive type. All samples were examined twice, 2 weeks apart by the same operator, and intraexaminer agreement was determined (Figures 8 and 9). Selected samples were prepared for scanning electron microscope (SEM) examination (Scanning electron microscope VEGA 3 XMU, Tescan, Brno, Czech Republic). Each specimen was gold coated with a sputter coater (SC7620 Mini Sputter Coater SC7620, Quorum Technologies, Lewes, United Kingdom) and mounted to the coded brass stubs to be examined at 100x magnification (Figures 10(a) and 10(b)).

Data obtained in terms of load failure and percentage of different failure modes were imported to Statistical Software (SPSS version 20.0, IBM Corp., New York, USA). Descriptive statistics and the Analysis of Variance test (ANOVA) at *P* ≤ 0.05 were used to determine the effect of failure loads among different groups. Differences between the groups were assessed by using the posthoc Tukey HSD test. In addition, the percentage of each mode of failure was calculated. The difference of failure between and within the 4 groups was revealed using Chi-square. The intraexaminer agreement was assessed using Kappa statistics.

## 3. Results

**Figure 11:**
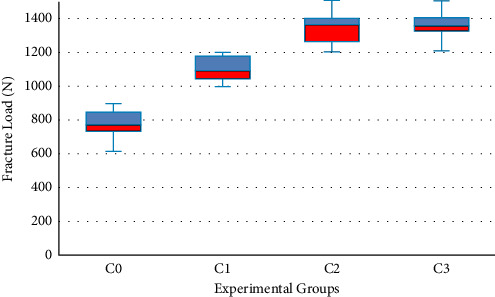
Boxplot of fracture load values measured in newtons (N).

The mean, standard deviation, minimum, and maximum fracture loads for the 4 groups are listed in Table 3 (Figure 11). The results of ANOVA statistics revealed that there were statistically significant differences in the fracture resistance between the experimental groups (*P* < 0.0001).

Multiple comparison between groups using the Tukey HSD test showed that there was a significant difference in the load to fracture among the 4 groups. Groups with palatal metal collar presented significantly higher failure loads when compared to the collarless group (*P* < 0.0001). However, the difference in failure loads between copings with 1.5- and 2- mm palatal metal collar height was not statistically significant (*P*=0.935). Group analysis of the mode of failure using Chi-square statistics revealed a nonsignificant difference in the mode of failure between the different groups (Table 4). All failures were located on the occlusal surface of the crown extending from the point of load application to the palatal surface of the crown.

Fracture surface examination of selected specimens using SEM detected reflective regions or mirrors surrounding the indentation area and the presence of markings including hackles and wake hackles were extending towards the margins of the porcelain veneer (Figures 10(a) and 10(b)) [27].

Kappa statistics demonstrated total agreement between both recorded readings by the same operator with 100% accuracy.

## 4. Discussion

The present study demonstrates that single metal-ceramic crowns with a palatal metal collar require a higher load to fracture than collarless single metal-ceramic crowns. Moreover, the study shows that as the height of the palatal metal collar increases, the fracture resistance of the metal-ceramic crown increases. Therefore, the null hypothesis was rejected.

Advancements in digital dentistry facilitated the fabrication of metal-ceramic restorations using a sintered metal substructure and pressed ceramic application to reduce the overall procedure cost and time. Until date, there are no clinical trials evaluating the reliability of the metal-ceramic bond established between sintered CoCr metal substructure and pressed ceramic in metal-ceramic restorations. However, it has been shown in laboratory experimental studies that the materials were compatible and resulted in an adequate metal-ceramic bond with reliable shear bond strength [31]. In Implant-prosthetic rehabilitations, CAD/CAM is used in the fabrication of custom abutments. These abutments are fabricated from titanium and/or zirconia which, as with metal-ceramic crowns, have numerous properties that can additionally enhance the esthetic outcome of anterior restorations [32].

An important finding of the present study is that none of the experimental models failed after exposure to thermocycling with no detected cracks or fractures. However, the longevity of a restoration is affected by the hostile environment resulting from temperature fluctuations and chemo-mechanical and microbiological influences [33]. Specimens in the present study were only subjected to thermal changes prior to fracture resistance testing, which only provides a partial indication to what occurs in the complex process of mastication. Nonetheless, exposure to thermal extremities simulates aging of the retentive crown system [34] and weakens the metal-ceramic bond [35]. This may result in the propagation of microcracks, [36, 37] which may fuse together to form a fissure that weakens the crown [38].

The failure loads recorded in the current study are about 3 to 5 times higher than the forces normally exerted in the premolar region (300 N) [39]. The maximum bite force for individuals is not constant and influenced by several factors such as the gender, age, jaw biomechanics, reflex mechanism, existing occlusion, and recording method [40]. In addition, oblique or horizontal forces produced during parafunctional mandibular movements might result in loads up to 6 times higher than the average biting force [41].

The results of the present study are in accordance with the findings of Michalakis et al. [9] and Goodacre et al. [15]. However, these studies did not specify the dimensions of the metal collar in terms of height. The findings of the current study contradict the findings of Gardner et al., [7] this contradiction could be due to the differences in the material and methods.

Failure analysis using SEM indicates that the fracture originated from the indentation area and propagated toward the margins of the porcelain veneer fracture [27, 42]. This agrees with the findings of Lorenzoni et al. [27]. However, in the current study, failure of metal-ceramic restorations included actual porcelain veneer fracture, whereas the previous study exhibited field damage through inner and outer cone crack formation without actual porcelain veneer fracture [27].

The use of natural teeth would make the experiment closer to the clinical scenario. However, extracted natural human teeth exhibit a large variation in age, size, shape, and quality, which would introduce unpredictable confounding variables. Moreover, storage conditions and timing after extraction of individual extracted human teeth can affect both the load required for fracture and the failure mode [9, 33]. In addition, fractures of teeth occurred when naturally extracted human teeth were cemented with metal-ceramic crowns and then loaded [33]. Previous studies demonstrated that the fracture pattern that occurred in natural teeth in laboratory studies was different than patterns detected in clinical studies [5, 33, 43]. In the current study, the metal used to fabricate the metal teeth analogs has a different modulus of elasticity (200 GPa) than the modulus of elasticity of dentin (14.7 GPa). Hence, the actual force distribution occurring on crowns cemented on natural teeth differs from the force distribution on those cemented on metal tooth analogs [9]. In addition, differences exist between the bonding properties of metal-ceramic crowns to chrome-cobalt alloys and dentin. A previous in vitro study concluded that bonding to dentin significantly increased the load required for subsequent failure [44].

Various factors that simulate the clinical situation such as the application of finger pressure during cementation procedures can be considered a limitation since it was not standardized. Limitations associated with the present laboratory study make it difficult to obtain comparable clinical results. Therefore, long-term prospective clinical studies are essential to confirm these findings.

## 5. Conclusions

Within the limitation of this study, the following conclusions were drawn:

1. The palatal metal collar improved the fracture resistance of metal-ceramic single crowns.
2. As the height of the palatal collar increased, the load required for fracture increased. However, there was no statistically significant difference between 1.5-mm and 2-mm metal collar height in influencing the fracture resistance.
3. None of the metal-ceramic crowns failed after exposure to 5000 cycles of thermocycling with abrupt temperature fluctuations.
4. 75% of the samples that fractured demonstrated a combined adhesive-cohesive type of failure, whereas only 25% of the fractured samples were classified with a cohesive failure.
5. The failure loads for all groups were considerably greater than the average occlusal forces exerted in the premolar area in natural dentition.
6. Failure analysis indicates that the fracture originated from the indentation area and propagated toward the margins of the porcelain veneer fracture.

## Figures and Tables

**Table 1:** Materials with corresponding manufacturer.

Material	Manufacturer	Country of production
Dental model AG3	Frasaco	North Carolina, USA
Hydroise fast set putty	Zhermack	Badia Polesine, Italy
Ceramill map 400 scanner	Amann girrbach	Vorarlberg, Austria
Ceramill sintron CoCr	Amann girrbach	Vorarlberg, Austria
CAD/CAM ceramill motion 2 milling unit	Amann girrbach	Vorarlberg, Austria
Ceramill argotherm 2	Amann girrbach	Vorarlberg, Austria
NTI carbide finishing bur	Kerr	California, USA
Ceramill mind CAD software	Amann Girrbach	Vorarlberg, Austria
Basic classic dental sandblaster	Renfert	Illinois, USA
Crea ally bond	Creation wili geller	Meiningen, Austria
Programat EP 5010	Ivoclar vivadent	Schann, Liechtenstein
IPS-InLine® opaquer	Ivoclar vivadent	Schann, Liechtenstein
3D sprint ceramill software	Amann girrbach	Vorarlberg, Austria
Next dent cast	Amann girrbach	Vorarlberg, Austria
Next dent LC 3D print box	Amann girrbach	Vorarlberg, Austria
Cervical wax	Renfert	Illinois, USA
Bellavest SH investment material	BEGO	Bremen, Germany
IPS-InLine® POM	Ivoclar vivadent	Schann, Liechtenstein
BioSonic UC125 ultrasonic cleaner	Coltene/Whaledent	Altstatten, Switzerland
Dentsply finishing and polishing kit	Dentsply sirona	New York, USA
IPS Inline® glaze	Ivoclar vivadent	Schann, Liechtenstein
DMG O-bite	DMG	New Jersey, USA
Ketac cem aplicap luting cement	3M ESPE	Minnesota, USA
Incubator I	Memmert GmbH	Schwabach, Germany
Thermocycler THE-1100/THE-1200	SD mechatronik	Feldkirchen-Westerham, Germany
Leica EZ4 stereomicroscope	Leica	Wetzlar, Germany
Universal testing machine M350-5CT	Testometric	Rochdale, United Kingdom
Scanning electron microscope VEGA 3 XMU	Tescan	Brno, Czech Republic
Mini sputter coater SC7620	Quorum technologies	Lewes, United Kingdom
SPSS version 20.0	IBM corp.	New York, USA

**Table 2:** Composition of ceramill sintron CoCr (amann girrbach, vorarlberg, Austria) [29].

Ceramill sintron composition						
Material	Co	Cr	Mo	Si	Fe	Mn
Content (%)	66%	28%	<1%	<1%	<1%	<1%

**Table 3:** Descriptive statistics of fracture load values (N).

Groups (mm)	Minimum (N)	Maximum (N)	Mean (SD) (N)
Palatal collar height at 0 mm (C0)	612.6	895	775.6^a^ (83.43)
Palatal collar height at 1.0 mm (C1)	995.6	1199.2	1103.783^b^ (75.26)
Palatal collar height at 1.5 mm (C2)	1201.2	1509.1	1344.883^c^ (96.09)
Palatal collar height at 2.0 mm (C3)	1208.3	1505.5	1366.100^c^ (96.49)

Different superscript letters indicate significant difference at *P* ≤ 0.05.

**Table 4:** Number and percentage (%) of failure mode.

Failure mode	Cohesive	Mixed adhesive-cohesive
Number	12	36
Percentage	25%	75%

## Data Availability

The readers can access the data supporting the conclusions of the current study.
